# The evaluation of a surgery and the short-term benefits of a new active bone conduction hearing implant - the Osia®^☆^

**DOI:** 10.1016/j.bjorl.2020.05.021

**Published:** 2020-07-04

**Authors:** Wojciech Gawęcki, Renata Gibasiewicz, Joanna Marszał, Magdalena Błaszczyk, Maria Gawłowska, Małgorzata Wierzbicka

**Affiliations:** a Poznań University of Medical Sciences, Department of Otolaryngology and Laryngological Oncology, Poznań, Poland; b Medicus sp. z o.o., Wrocław, Poland; c University of Silesia in Katowice, Katowice, Poland

**Keywords:** Bone conduction hearing aid, Piezoelectric actuator, Active hearing system, Osia®, Baha®Attract

## Abstract

**Introduction:**

Modern medicine offers a wide spectrum of different hearing devices, and bone conduction implants can be found among them.

**Objective:**

The presentation of the outcomes of the implantation of a new active bone conduction hearing implant – the Osia®, and its comparison with the well-known passive transcutaneous system – the Baha® Attract.

**Methods:**

Eight adult patients with bilateral mixed hearing loss were randomly divided into two groups. Group 1 was implanted with the Osia®, and group 2 was implanted with the Baha® Attract. The details of the surgery were analyzed, along with the functional and audiological results.

**Results:**

In all the cases, the surgery was successful, and the healing uneventful. In both groups, it was observed that pure tone audiometry and speech audiometry in free field improved significantly after the implantation (mean gain in pure tone audiometry for the Osia group 42.8 dB SPL and for the Baha group 38.8 dB SPL). In the Osia group, the results after the surgery were much better than with the Baha® 5 Power processor on the Softband. The patients implanted with the Osia® evaluated the quality of their hearing as being superior to those implanted with the Baha® Attract. There was an evident improvement in the abbreviated profile of hearing aid benefit questionnaire and in the speech, spatial and qualities of hearing scale for both systems. In the abbreviated profile of hearing aid benefit, changes were more evident in the Osia group (in global score 49% vs. 37.2%).

**Conclusion:**

Implantation of the Osia® is an effective treatment option for the patients with bilateral mixed hearing loss. The surgery is safe but more complex and time-consuming than the Baha® Attract implantation. The preliminary audiological results as well as the overall quality of life indicate that the Osia® is a better solution than the Baha® Attract. However, future studies should be carried out to make further observations in a larger group of patients, and with longer follow-up.

## Introduction

Modern medicine offers a wide spectrum of different hearing devices for hearing impaired patients – both hearing aids and many implantable systems. Currently, four types of hearing implants are available: cochlear, bone conduction, middle ear and auditory brainstem implants.^1^, ^2^, ^3^, ^4^

Bone conduction implants (BCIs) or bone-anchored hearing aids (BAHAs) are well-established solutions used in treatment of patients with unilateral and bilateral, mixed, and conductive hearing loss, as well as those with single-sided deafness. The first implantation was described by Tjellström and Granström in 1977^5^ and since then, many different systems have been developed.^6^

The most popular systems are known as passive bone conduction systems, which consist of an implantable part being gradually osseointegrated with a bone, an external sound processor with a transducer, and a connecting part – the skin penetrating abutment (in percutaneous solutions) or system of magnets (in transcutaneous devices). Both systems are very helpful in the above-mentioned patients, however, they are not ideal and have some disadvantages and limitations.^6^

The percutaneous BAHA is a skin penetrating solution, which enables direct and high-quality sound transmission. However, it breaks the continuity of the skin barrier, so it requires lifelong daily hygienic care. What is more, it causes the risk of local skin complications including infections, skin overgrowth and sometimes even implant loss.^7^, ^8^ The frequency of recurrent soft tissue reactions and infections around the abutment were reported to be found in 8%−59% of the cases; implant loss – in 8.3%; and the need for revision surgery − in 5%−42%.^7^ Additionally, the cosmetic effect is not optimal, and some patients who might benefit from the system, decline implantation.^9^ Currently, there are two percutaneous systems commercially available: the Baha® Connect (Cochlear Ltd, Australia) and the Ponto® (Oticon, Denmark).

The transcutaneous BAHA, in contrast to the previous one, uses magnetic attraction force, and leaves no permanent skin defect. Thus there are no hygienic problems, and the aesthetic effect is good. However, the quality of sound can be limited due to the sound attenuation by the intact skin between the magnets.^9^, ^10^ Moreover, the permanent pressure to the skin can lead to redness or pain over the magnet, and sometimes even to soft tissue necrosis.^11^, ^12^ Presently, there are two different systems available on the market: the Sophono® (Medtronic, USA) and the Baha® Attract (Cochlear Ltd, Australia).

In recent years, a few systems, known as active bone conduction systems, have become available.^6^, ^13^ With their use, the problem of skin discontinuity and soft tissue attenuation can be avoided, since in these solutions, a transducer is located directly in/on a patient’s bone, and an external processor (without a transducer) is attached to the head by magnetic attraction force. The first such an electromagnetic device − the Bonebridge® (Medel, Austria) was introduced to the market a few years ago, and another electromagnetic one – the Sentio (Oticon, Denmark) is currently in a regulatory process for CE (European Conformity) marking. Furthermore, in 2019, a new powerful active bone conduction system with piezoelectric transducer – the Osia® from Cochlear Ltd, received CE, and was available as an early market release to 8 selected European clinics, including our department.

The aim of this study was to present the outcomes of the implantation of the new Osia® system (primary goal), and to compare it with the well-known Baha® Attract (secondary goal).

## Methods

### Study design and patients’ characteristics

The prospective study was conducted between June and December 2019. Eight adult patients with bilateral mixed hearing loss, who were candidates for the bone conduction device implantation, were enrolled and randomly divided into two groups. Group 1 (n = 4) was implanted with the Osia®, and Group 2 (n = 4) was implanted with the Baha® Attract. The detailed descriptions of the patients are presented in Table 1.

**Table 1:** Patients’ characteristics.

	Group 1 – the Osia group	Group 2 – the Baha group
Implanted device	The Cochlear™ Osia®	The Cochlear™ Baha® Attract
Age (years)	Mean 58 (min. 38, max. 76)	Mean 51 (min. 26, max. 65)
Hearing loss	Bilateral mixed	Bilateral mixed
Air conduction in an ear at the side of the implantation – PTA (dB HL)	80	67.5
Bone conduction in an ear at the side of the implantation – PTA (dB HL)	43.5	38.8
Etiology of hearing loss	Bilateral chronic otitis media – 3	Bilateral chronic otitis media – 2
	Bilateral otosclerosis – 1	Bilateral otosclerosis –1
		Treacher Collins syndrome – 1
Side of the implantation	Right – 1	Right – 3
	Left – 3	Left – 1

PTA (dB HL), Pure Tone Average for 0.5, 1, 2, and 4 kHz (decibels hearing level).

### The Osia® system

**Figure 1:**
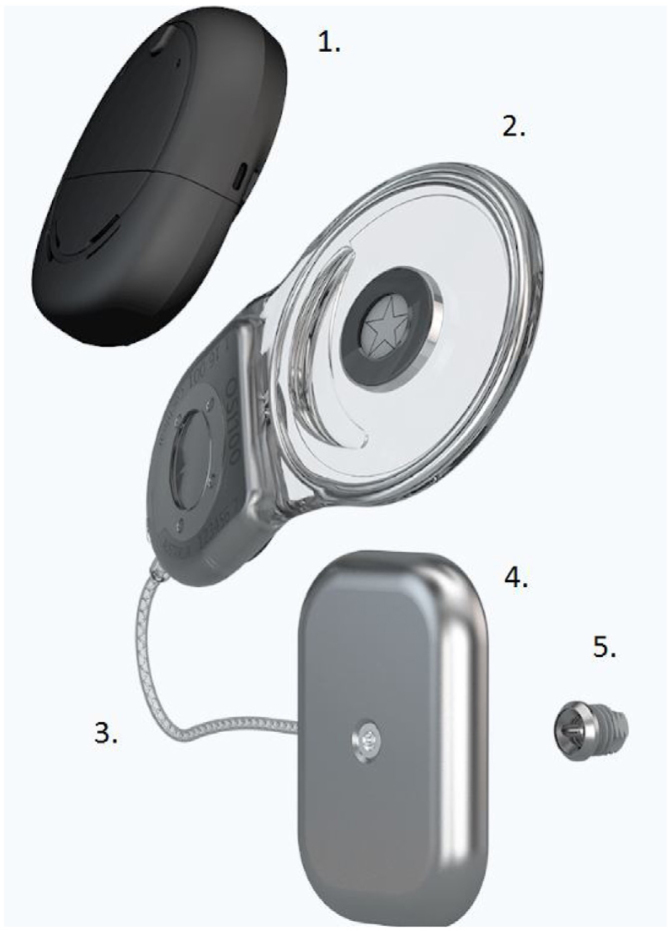
The Cochlear™ Osia® System. 1 – A sound processor; 2 – A receiver-stimulator module; 3 – A connecting cable; 4 – A piezoelectric transducer; 5 – A BI300 implant. Images courtesy of Cochlear Limited©. Cochlear Limited 2020. All rights reserved.

The new active piezoelectric bone conduction system evaluated in this study is composed of implantable parts (a typical BI300 bone conduction implant, a piezoelectric transducer attached to an implant, and a receiver-stimulator module, similar to that in a cochlear implant) and an external sound processor (Fig. 1).

### Evaluated parameters

The following parameters were evaluated, and might be presented as three categories:

1 Surgery and healing: type of anesthesia, time of a surgery, length of incision, implant position, soft tissue reduction, bone polishing, bleeding from bone at the site of implantation, or any additional problems and difficulties during a surgery, as well as healing process, pain and numbness.
2 Audiological results:
a) Pure tone audiometry, performed (1) with headphones (air and bone conduction), and (2) in free field: first, before the surgery with the Baha® 5 Power sound processor on the Softband, and then, after the surgery with an implanted device (the Baha® Attract or the Osia® system); free field thresholds were measured using warble tones presented from a loudspeaker that was positioned 1 m in front of a patient,
b) Speech audiometry (a Polish monosyllabic word test), performed (1) with headphones, and (2) in free field: first, before the surgery, with the Baha® 5 Power sound processor on the Softband, and then, after surgery with implanted device (the Baha® Attract or the Osia® system), both in quiet and noise (the speech of 50 dB, 65 dB and 80 dB SPL was presented from a loudspeaker that was positioned 1 m in front of a patient; the noise of 55 dB SPL was presented from a loudspeaker placed behind a patient),
c) Direct bone conduction through an implanted device (BC in situ),
d) Patients’ subjective evaluation of the quality of sounds after the implantation of a device – 4 parameters were evaluated according to a scale ranging from 1 (the worst) to 5 points (the best): sound loudness, sound distinction, hearing of one’s own voice and reverberation.
3 The quality of life benefits: the APHAB (the Abbreviated Profile of Hearing Aid Benefit) and the SSQ (the Speech, Spatial and Qualities of Hearing Scale) questionnaires.

The audiological evaluation as well as the assessment of patients’ quality of life was performed 3 months after the surgery. All the audiological tests were performed with the Otometrics Madsen Astera in a soundproof room. The investigation was approved by the local Ethics Committee [decision number 42/19].

## Results

### A surgery and healing

In all the cases, the surgery was successful, and the healing uneventful. However, the Osia® surgery required general anesthesia, and was much more complex, in comparison to the Baha® Attract, as a longer skin incision and more bone work were needed (including bone polishing in the area of a BI300 implant in all the cases, and bone bed preparation for the receiver/stimulator). Three months after the surgery, the operated area was free of pain in all the cases, but in 1 Osia patient and 1 Baha patient, skin sensitivity was still limited.

**Figure 2:**
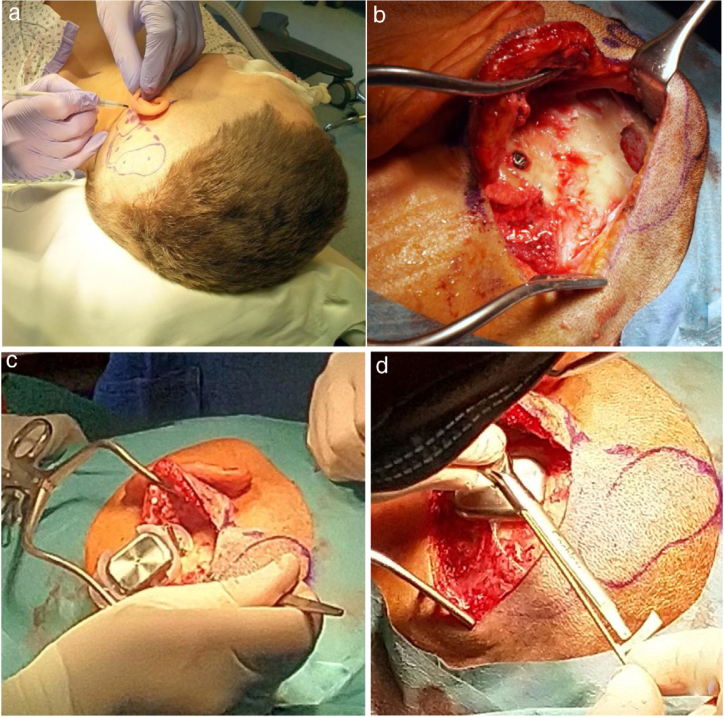
The Osia® surgery: (a) the marked place for the device, (b) the BI300 implant in place, reduced bone around, prepared subperiosteal pocket and bone bed, (c) the device partially in place (the piezoelectric transducer still not connected to the BI300 implant) − intraoperative measurements, (d) the whole device in place (the piezoelectric transducer connected to the BI300 implant).

The details are presented in Table 2, and the pictures from the Osia® surgery are presented in Fig. 2.

**Table 2:** The details of the surgery and healing, for the Osia® and the Baha® Attract.

	The Osia group (n = 4)	The Baha group (n = 4)
Anesthesia	General	Local
An average surgery time (min)	119 (110 − 130)	51 (40 − 67)
An average incision length (mm)	91 (85 − 100)	55 (45 − 65)
An average distance: implant – the center of the ear canal (mm)	40 (38 − 45)	69 (65 − 75)
Soft tissue reduction (n)	1	1
Bone reduction (n)	4	1
Bleeding from a bone (n)	1	1
Other problems	In 1 case, an air cell in the place of the hole for an implant; a new hole was created more posteriorly	No
Healing	Uneventful	Uneventful
Pain 3 months after the surgery (n)	0	0
Numbness 3 months after the surgery (n)	1	1

n, Number of patients.

### Audiological benefits

#### Pure tone audiometry

**Figure 3:**
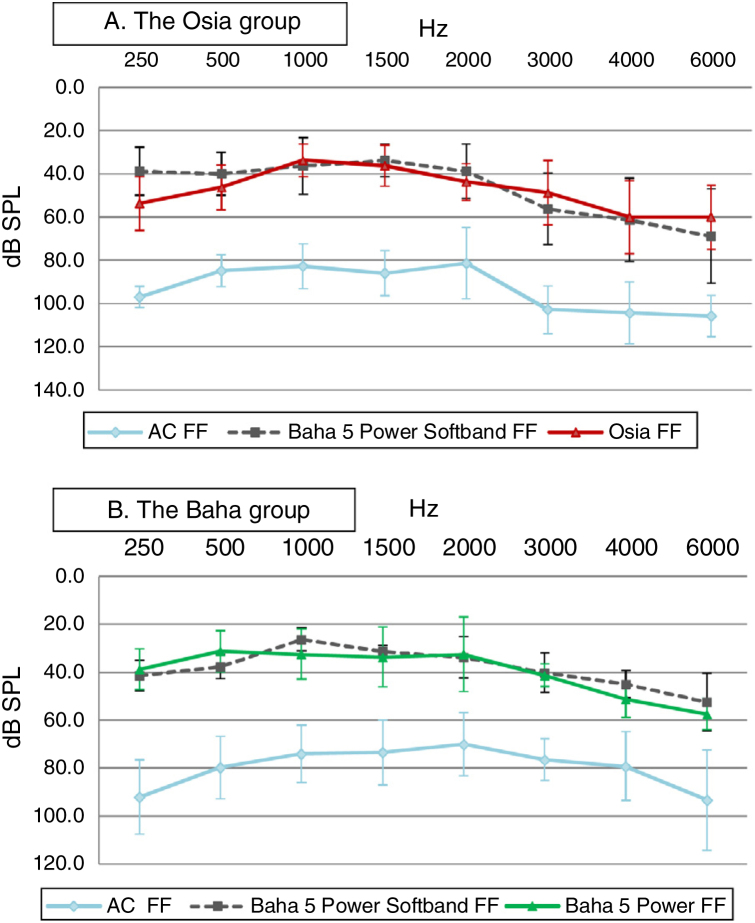
The results of pure tone audiometry in free field with an implanted device in comparison to the preoperative results − unaided and with the Baha® 5 Power processor on the Softband: (A) for Osia group, (B) for Baha group.

In both groups, significant improvement was observed in terms of pure tone audiometry in free field with an implanted device, in comparison to unaided hearing. The mean gain in PTA for the Osia group was 42.8 ± 4.9 dB SPL, and for the Baha group 38.8 ± 8.5 dB SPL. In both groups, the obtained results were comparable to those with the Baha® 5 Power processor on the Softband (Fig. 3).

#### Speech audiometry

**Figure 4:**
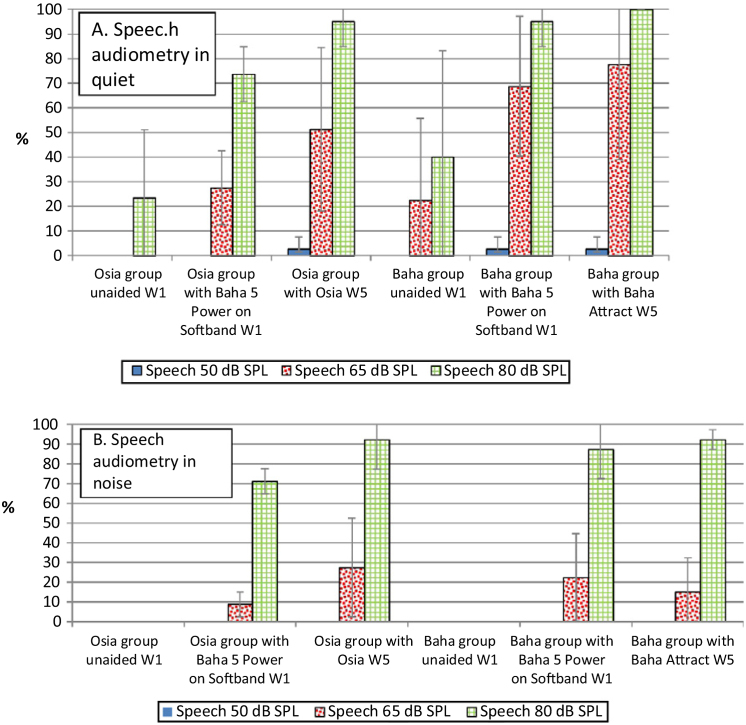
The results of speech audiometry in free field for both groups (mean value with a standard deviation − SD): A, in quiet and B, in noise (W1 – before the surgery, W5 – 3 months after the surgery).

In comparison to the unaided situation, it was observed that speech understanding in free field, both in quiet and noise, improved significantly after the implantation of a device in both groups. For silent and loud speech (50 dB and 80 dB), both in quiet and noise, the results for the Osia and the Baha group were similar. Nevertheless, for medium speech (65 dB), the results in quiet were better for the Baha group, whereas in noise, they were better for the Osia group. Generally, after the surgery, the results for the Osia group were better, in comparison to those with Baha® 5 Power processor on the Softband. The results for the Baha group after the implantation and with the Softband were similar (Fig. 4).

#### Direct bone conduction

**Figure 5:**
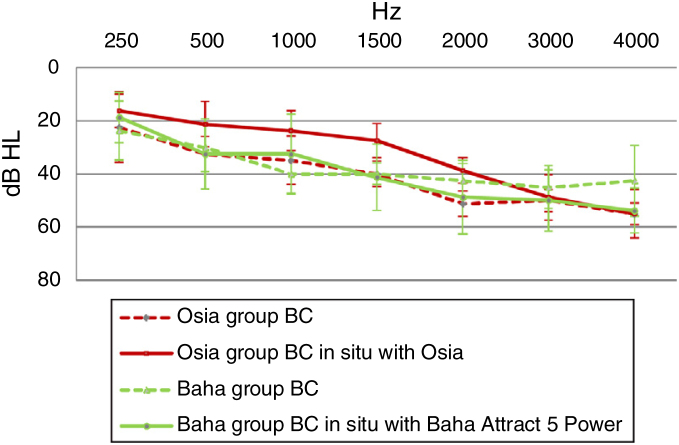
The difference in bone conduction between the measurements in situ with an implanted device, and those performed preoperatively by using pure tone audiometry with headphones − for the Osia group and the Baha group.

In the Osia group, for most sound frequencies, the results of the Bone Conduction (BC) in situ measurements with an implanted device were better, in comparison to the preoperative levels. In the Baha group, the respective results were similar or even worse after the surgery (Fig. 5).

#### The subjective quality of hearing

**Figure 6:**
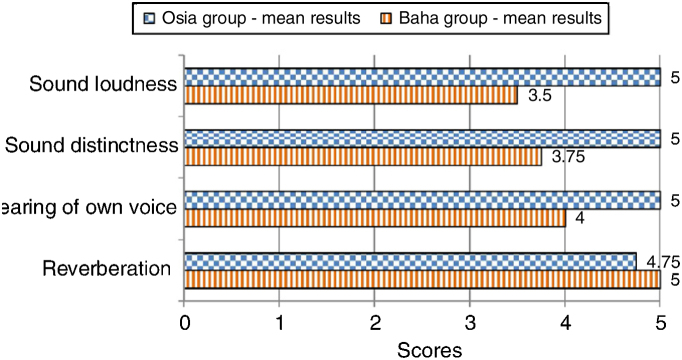
The quality of hearing – subjective patients’ opinion − every parameter evaluated with points ranging from 1 (the worst) to 5 (the best).

The patients evaluated four aspects of the quality of their hearing: sound loudness, sound distinctness, hearing of their own voice, and reverberation. The subjects implanted with the Osia® marked three out of four above-mentioned aspects of hearing higher, in comparison to the patients implanted with the Baha® Attract. In the case of the remaining one aspect, the results were similar for both groups. The details are presented in Fig. 6.

### Quality of life results

**Figure 7:**
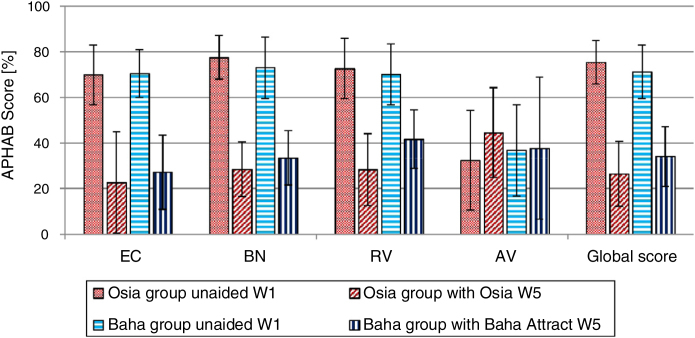
The benefits of the Osia® and the Baha® Attract implantation – the APHAB scale – a mean value with SD (W1, before surgery; W5, 3 months after surgery; EC, Ease of communication; BN, Background Noise; RV, Reverberation; AV, Aversiveness; Global score, a mean of EC, BN and RV).

**Figure 8:**
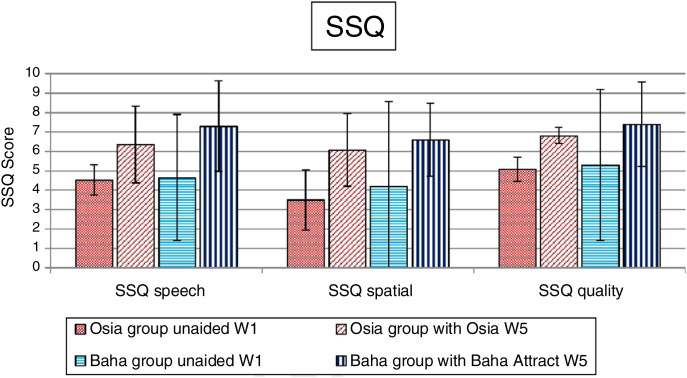
The benefits of the Osia® and the Baha® Attract implantation – the SSQ scale – a mean value with SD (W1 – before surgery, W5 – 3 months after surgery).

The number of hearing problems evaluated by the APHAB scale in different acoustical situations was significantly reduced after the implantation, for both systems. The changes were more evident in the Osia group (reduction of problems in global score 49.0%±16.1% vs. 37.2%±15.2%). The details are presented in Fig. 7. Similarly, after the implantation, an evident improvement was observed in terms of speech, spatial and the quality of hearing, measured by the SSQ scale, for both systems, and the results were slightly better for the Baha group. The details are presented in Fig. 8.

## Discussion

Currently, the place of bone conduction devices in hearing restoration is well-established, and the benefits of these solutions in well-qualified cases are not in dispute. However, the available systems are not flawless, and the choice of an optimal option is not usually simple.^6^ For many years, getting a suitable audiological gain has not usually been consonant with good esthetic and hygienic results. Moreover, the most traditional form of bone conduction device with skin-penetrating abutment remained the only effective solution for the patients with a significant sensorineural component of hearing loss. However, devices known as active bone conduction systems, were created in order to combine the benefits of direct transmission of vibration produced by a device, favorable esthetic and hygienic effect after surgery, and furthermore to reduce the size of a visible part of the system.^6^, ^13^ One of them – the new piezoelectric Osia® − was evaluated in this study. To the best of our knowledge, until the time of the preparation of this manuscript, there had been no published papers concerning surgery and benefits of the above-mentioned device.

Our study showed that an Osia® surgery is safe, but more complex and much more time-consuming than a Baha® Attract surgery.

What is more, we found that there is an evident audiological benefit after an Osia® implantation, and a significant improvement in pure tone audiometry and speech audiometry, as well in quiet as in noise, when compared to the unaided conditions. Moreover, the audiological gain of our patients after the Osia® implantation was compared to the performance with the Baha® 5 Power on the Softband before the surgery and we found an evident improvement in speech audiometry, both in quiet and noise. In our research, the advantages of the Osia® compared to the Baha® Attract were also discussed. We did not find any evident differences between both groups, in terms of free field pure tone audiometry or speech audiometry improvements. However, there were some differences in the age and preoperative hearing status before the surgery in our randomized groups. In comparison to the Baha group, the mean age in the Osia group was higher, and the initial hearing performance was worse. Thus, a similar hearing improvement for both devices indicates that the Osia® is a better choice. Moreover, the patients’ subjective evaluation of the quality of their hearing with both devices confirms this observation. Additional audiological evaluation of our patients showed better results of bone conduction measured with the Osia® device (BC in situ) than, the one measured preoperatively with bone headphones, which resulted from the direct stimulation of the bone without attenuation by the skin. In the Baha group, such difference was not observed, which is obvious. Moreover, according to our study, the subjective evaluation of four aspects of the quality of hearing indicates that the Osia® is a better solution than the Baha® Attract.

We also observed an evident improvement in quality of life after the Osia® implantation, measured by the APHAB and the SSQ scale. The gain in the reduction of hearing problems in different acoustical situations, evaluated by the first questionnaire, was more evident in the Osia group than in the Baha group, and the results of the second questionnaire were slightly better for the Baha group.

Our studies have some limitations, such as a small number of implanted patients, short time of observation, and the differences in age and preoperative hearing status before the surgery in our randomized groups.

## Conclusion

The Osia®, a new active bone conduction implant, is an effective treatment option for the patients with bilateral mixed hearing loss. The surgery is safe, but more complex and time-consuming than the Baha® Attract implantation. In comparison to the Baha® Attract, the early audiological and quality of life results, observed in our small groups of patients, indicate that the Osia® is a better solution. The exceptionally noteworthy results are: significant improvement in speech understanding after the Osia® implantation, in comparison to the Baha® 5 Power on the Softband, the subjective patients’ evaluation of the quality of hearing, and the obtained gain in the reduction of hearing problems in different acoustic situations. Nevertheless, further observations should be carried out in groups of cases, and with longer follow-up.

The study was not supported by Cochlear Ltd.

## Conflicts of interest

The authors declare no conflicts of interest.
